# Aldehyde dehydrogenase 1 expression correlates with the invasion of breast cancer

**DOI:** 10.1186/s13000-015-0301-5

**Published:** 2015-06-13

**Authors:** Hong Pan, Naping Wu, Yaoyu Huang, Qin Li, Chenghao Liu, Mengdi Liang, Wenbin Zhou, Xiaoan Liu, Shui Wang

**Affiliations:** Department of Breast Surgery, The First Affiliated Hospital with Nanjing Medical University, 300 Guangzhou Road, 210029 Nanjing, China; Department of Breast Surgery, The Third Affiliated Hospital with Suzhou University, 185 Juqian Street, Changzhou, 213000 China

**Keywords:** ALDH1, Breast cancer, DCIS, Invasive cancer

## Abstract

**Background:**

Aldehyde dehydrogenase 1 (ALDH1) is an important marker of tumor-initiating cells. We aimed to investigate ALDH1 expression in benign breast disease and human breast cancer of different histologic stages.

**Methods:**

Immunohistochemical staining of ALDH1 was applied to 21 cases with benign breast diseases, 47 ductal carcinoma *in situ* (DCIS) cases, 62 cases diagnosed with invasive cancer with extensive intraductal component (EIC), and 58 cases diagnosed with invasive cancer without EIC.

**Results:**

ALDH1 was expressed in tumor cells in 61.0 % of 164 breast cancer cases, which was higher than that in benign breast disease (3/21) (*P* < 0.001). Of these 167 breast cancer cases, a significantly higher rate (54/58) of intratumoral ALDH1 expression was observed in invasive cancer without EIC cases than that in DCIS cases (19/46, one case not available) and invasive cancer with EIC cases (27/60, two cases not available) (*P* < 0.001). Interestingly, a significantly higher rate (22/48) of intratumoral ALDH1 expression in invasive component was observed than that in *in situ* component (7/48) in the same tumor (*P* = 0.001). In 47 DCIS cases, no significant association was observed between ALDH1 positivity and any clinicopathological parameter (all *P* > 0.05). However, ALDH1 positive invasive breast cancers were significantly more likely to be with large tumor size (*P* = 0.001), high grade (*P* < 0.001), and high Ki67 expression (*P* = 0.009).

**Conclusions:**

ALDH1 may play an important role in the invasion of breast cancer, and may be associated with aggressive phenotypes of breast cancer.

**Virtual slides:**

The virtual slide(s) for this article can be found here: http://www.diagnosticpathology.diagnomx.eu/vs/1608671725154947.

## Background

Breast cancer is a worldwide malignant disease. Recent studies [1–3] suggest that this disease is driven by a subpopulation of breast cancer cells, called breast tumor-initiating cells (BT-ICs), which have been identified with the capacity for self-renewal and the ability to generate different cell types [4]. BT-ICs bear the phenotype of CD44^+^/CD24^-^ [5], and aldehyde dehydrogenase 1 (ALDH1) is also an important marker of BT-ICs [6]. Ginestier and colleagues have found that ALDH1 may be a better marker of BT-ICs than CD44^+^/CD24^-^in immunodeficient mice [6].

Previous studies [1, 6-8] have found ALDH1 positive cancer cells may be associated with aggressive phenotypes and poor clinical outcomes in breast cancer patients. Park and colleagues have attempted to investigate the role of ALDH1 in the progression of breast cancer [9]. They have found ALDH1 positive cells were more frequent in basal-like and HER2+ than in luminal tumors, but no significant difference of ALDH1 expression was observed in four histologic groups (invasive ductal carcinoma (IDC), IDC with ductal carcinoma *in situ* (DCIS), DCIS with microinvasion, and pure DCIS). However, the ALDH1-positive rate in IDC seems higher than that in DCIS in another study [1]. The previous results are discordant, and the cutoffs of positive ALDH1 in different studies are different [1, 6-9].

Furthermore, the normal mammary stem-like cells can also be identified by the expression of ALDH1 [10–12]. Isfoss and colleagues have found a positive association between the frequency of ductular ALDH1 positive cells and several breast cancer risk factors in histologically normal breast tissue [12], which supports previous evidence that ALDH1 may play a role in the development of breast cancer.

In this study, ALDH1 expression was investigated in benign breast disease and human breast cancer of different histologic stages (DCIS, invasive cancer with extensive intraductal component (EIC), and invasive cancer without EIC). Furthermore, the associations between ALDH1 positivity and clinicopathological parameters were also determined.

## Methods

### Patients

The present study was approved by the ethics committee of The First Affiliated Hospital with Nanjing Medical University. Written, informed consent was given by the patients for their information to be used for research. This study was also in compliance with the Helsinki Declaration.

From January 2010 to December 2011, operable breast cancer patients, diagnosed with DCIS, invasive cancer with EIC, and invasive cancer without EIC by using resection specimen, were screened in our hospital. The exclusion criteria included the following: (a) patients treated with neoadjuvant chemotherapy; (b) patients diagnosed with breast cancer by using core biopsy; (c) not enough specimens for further pathological analysis. At last, 167 consecutive breast cancer patients were recruited in this study, including 47 DCIS cases, 62 cases diagnosed with invasive cancer with EIC, and 58 cases diagnosed with invasive cancer without EIC. Furthermore, specimens of 21 benign breast diseases (mastopathy, hyperplasia, and fibroadenoma in this study) were included in this study.

### Pathology

The pathology was reviewed by two experienced pathologists independently. Disagreements were resolved with consensus opinion. The specimens were paraffin-embedded for histopathological examinations. Then, 4 μm histological sections were cut and stained with hematoxylin and eosin (H&E). DCIS with microinvasion ≤ 1 mm was also considered as the DCIS category in our study. EIC was defined as positive if the proportion of DCIS was greater than 25 % of the whole tumor in pathologic sections [13, 14].

Immunohistochemical (IHC) analyses on paraffin-embedded material were used to determine the status of estrogen receptor (ER), progesterone receptor (PR), Her2, and Ki67. The status of ER, PR, Her2 and Ki67 were determined as described previously [15, 16]. Low Ki67 expression was defined as positive Ki67 staining ≤14 % in pathologic sections, while high Ki67 expression was defined as positive Ki67 staining >14 %. Patients with invasive breast cancer were categorized into triple negative and non-triple negative breast cancer in this study. Triple negative breast cancer was defined as ER, PR and Her2 negative.

ALDH1 status was also determined by IHC. IHC analyses were performed on 4 μm, formalin-fixed, paraffin-embedded slides from breast cancer tissues. Paraffin-embedded tissue sections were deparaffinized, rehydrated, rinsed, and immersed in 10 mM sodium citrate (pH 6.0) for antigen retrieval under high pressure in a pressure cooker for 3 minutes. After treated with methanol containing 3 % hydrogen peroxide for 10 min to block endogenous peroxidase activity, the slides were incubated with mouse monoclonal antibody directed against human ALDH1 (aa 7–128, diluted 1:100; Becton, Dickinson and Company) for 1 hour at 37 °C. After washing, sequential incubations were performed with horseradish peroxidase (HRP) conjugated antibodies (Invitrogen) for 30 min at room temperature. The stain was visualized using DAB Plus (Dako) and hematoxylin counterstain. Tumor presenting at least one ALDH1-positive cancer cell was considered as an ALDH1-positive tumor, and stroma presenting at least one ALDH1-positive stromal cell was considered as an ALDH1-positive stroma [1, 6].

### Statistical analysis

In our study, median, percentiles and range were analyzed for continuous variables. The variables in this study included: age at diagnose, pathology, tumor size, axillary node status, tumor grade, ER, Her2, Ki67, and molecular subtype. Differences between the subgroups with regard to above variables were examined using Fisher’s exact test or chi-square test. All *P*-values were two-tailed with 5 % significance levels. All statistical analyses were performed using STATA version 11.0 (Computer Resource Center, America).

## Results

### Baseline characteristics

167 patients with different stages of breast cancer were enrolled in the present study (Table 1), including 47 DCIS cases, 62 cases diagnosed with invasive cancer with EIC, and 58 cases diagnosed with invasive cancer without EIC. The median patient age was 48 years (range, 20-82 years). Of these 167 patients, 58 (34.7 %) were found with axillary node involved, and 114 (68.3 %) were diagnosed with ER positive breast cancer. Of 120 invasive cancer patients, 27 (22.5 %) were Her2 overexpressed.

**Table 1:** Characteristics of included breast cancer patients

Variables	Number (%)
Age (y)	
≤50	94 (56.3 %)
>50	73 (43.7 %)
Pathology	
DCIS	47 (28.1 %)
Invasive cancer	120 (71.9 %)
Tumor size^a^	
≤2 cm	55 (45.8 %)
>2 cm	63 (52.5 %)
Not available	2 (1.7 %)
Axillary node status	
Negative	103 (61.7 %)
Positive	58 (34.7 %)
Not available	6 (3.6 %)
ER status	
Negative	53 (31.7 %)
Positive	114 (68.3 %)
Her2 status^a^	
Negative	93 (77.5 %)
Positive	27 (22.5 %)

^a^DCIS not included for analysis

### ALDH1 expression in benign breast disease and breast cancer

**Fig. 1:**
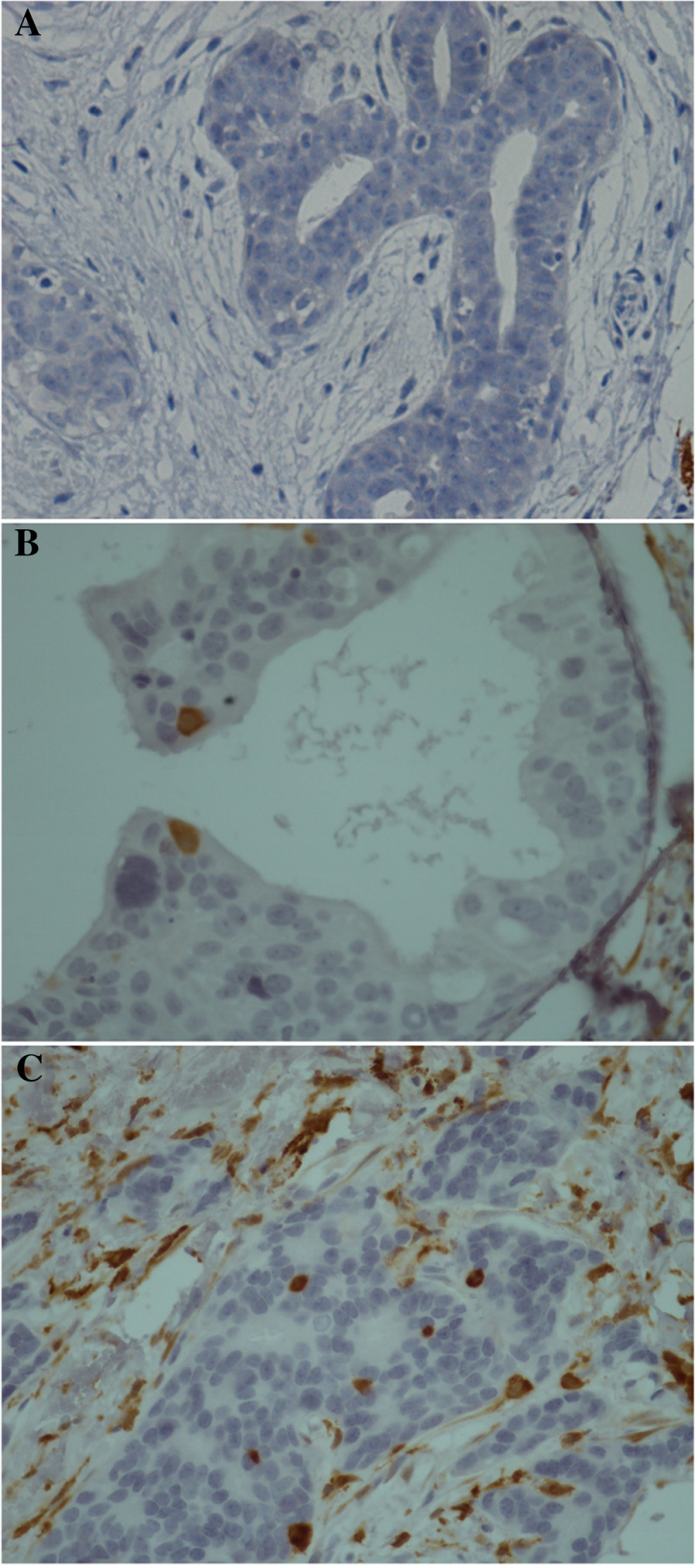
Immunohistochemical analyses of aldehyde dehydrogenase 1 (ALDH1) expression in benign breast disease (**a**), ductal carcinoma *in situ* (**b**), and invasive breast cancer (**c**)

ALDH1 protein expression was determined in benign breast disease and breast cancer tissues. Of 21 benign breast disease tissues, ALDH1 was only expressed in epithelial cells in 3 cases; while ALDH1 was expressed in tumor cells in 61.0 % (100/164, three cases not available) of 164 breast cancer cases (Fig. 1). A significant difference was observed between these two groups (*P* < 0.001).

Additionally, ALDH1 was expressed in stromal cells in 89.5 % (17/19, two cases not available) of benign cases; while ALDH1 was expressed in stromal cells in 92.0 % (150/163, four cases not available) of 164 breast cancer cases. There was no significant difference between these two groups (*P* = 0.702).

### ALDH1 expression in different stages of breast cancer

ALDH1 protein expression was determined in different stages of breast cancer, including DCIS, invasive cancer with EIC, and invasive cancer without EIC (Fig. 1). ALDH1 was expressed in stromal cells in 93.5 % (43/46, one case not available) of DCIS cases, 85.0 % (51/60, two cases not available) of invasive cancer with EIC cases, and 98.2 % (56/57, one case not available) of invasive cancer without EIC cases. The ALDH1 expression rates in stromal cells among these different stages of breast cancer were significantly different (*P* = 0.022, Table 2).

**Table 2:** ALDH1 expression in different stages of breast cancer

Pathology	Stromal ALDH1			Intratumoral ALDH1		
	Negative	Positive	P value	Negative	Positive	*P* value
DCIS	3	43	0.022	27	19	<0.001
Invasive + EIC	9	51		33	27	
Invasive	1	56		4	54	

Interestingly, ALDH1 was expressed in tumor cells in 41.3 % (19/46, one case not available) of DCIS cases, 45.0 % (27/60, two cases not available) of invasive cancer with EIC cases, and 93.1 % (54/58) of invasive cancer without EIC cases. A significant difference was observed in these three groups (*P* < 0.001, Table 2).

### Intratumoral ALDH1 expression in DCIS

The association between intratumoral ALDH1 expression and clinicopathological parameters of DCIS was investigated (Table 3). In these 47 DCIS cases, no significant association was observed between ALDH1 positivity and age (*P* = 0.528), tumor size (*P* = 0.951), grade (*P* = 0.812), ER status (*P* = 0.428), Her2 status (*P* = 0.536), or Ki67 (*P* = 0.667).

**Table 3:** Relationship between intratumoral ALDH1 expression and clinicopathological parameters of DCIS

Variables	Intratumoral ALDH1		
	Negative	Positive	P value
Age (y)			
<40	5	5	0.528
≥40	22	14	
Tumor size			
≤1.5 cm	10	8	0.951
>1.5 cm	13	10	
Grade			
I-II	9	7	0.812
III	15	10	
ER			
Negative	7	7	0.428
Positive	20	12	
Her2			
Negative	14	11	0.536
Positive	8	4	
Ki67			
≤14 %	10	9	0.667
>14 %	9	6	

### Intratumoral ALDH1 expression in invasive breast cancer

The relationship between intratumoral ALDH1 expression and clinicopathological parameters of invasive cancer was determined (Table 4). In these 120 invasive cancer cases, no significant association was observed between ALDH1 positivity and age (*P* = 0.243). A significantly higher rate (53/63) of intratumoral ALDH1 expression was observed in large tumors (> 2 cm) than that (30/54) in small tumors (≤ 2 cm) (*P* = 0.001). There was a trend that more intratumoral ALDH1 expressed in tumors with involved lymph nodes (43/57) than that without involved lymph nodes (38/60), but no significant difference was observed (*P* = 0.156). Importantly, a significantly higher rate (35/39) of intratumoral ALDH1 expression was observed in grade III tumors than that (36/63) in grade I-II tumors (*P* < 0.001).

**Table 4:** Relationship between intratumoral ALDH1 expression and clinicopathological parameters of invasive breast cancer

Variables	Intratumoral ALDH1		
	Negative	Positive	P value
Age (y)			
≤50	23	41	0.243
>50	14	40	
Tumor size			
≤2 cm	24	30	0.001
>2 cm	10	53	
Grade			
I-II	27	36	<0.001
III	4	35	
Axillary node status			
Negative	22	38	0.156
Positive	14	43	
ER status			
Negative	11	28	0.604
Positive	26	53	
Her2 status			
Negative	28	63	0.801
Positive	9	18	
Ki67			
≤14 %	15	21	0.009
>14 %	12	55	
Molecular subtype			
Triple negative	6	17	0.992
Non-triple negative	22	62	

No significant association was observed between intratumoral ALDH1 positivity and ER status (*P* = 0.604), or Her2 status (*P* = 0.801). Similarly, the frequencies of intratumoral ALDH1 positivity between triple negative and non-triple negative breast cancers were not significantly different (*P* = 0.992). The frequency of intratumoral ALDH1 positive tumors with high Ki67 expression was significantly higher than that with low Ki67 expression (*P* = 0.009).

### Intratumoral ALDH1 expression patterns of invasive and *in situ* components of the same tumor

To determine if the difference seen in the expression of some markers between invasive and *in situ* components may reflect a potential divergence in expression associated with *in situ* to invasive breast carcinoma progression, we examined the expression of ALDH1 in tumors with invasive and *in situ* areas present on the same slides. Of 62 invasive cancers with EIC cases, intratumoral ALDH1 expressions of both invasive and *in situ* components were available in 48 cases. ALDH1 was expressed in 22 cases in invasive component, and in 7 cases in the *in situ* component. A significant difference was observed in these two different components of the same tumor (*P* = 0.001). Of these 48 cases, ALDH1 was expressed in both invasive and *in situ* components in 7 cases. In these 7 cases, a higher percentage of ALDH1 positive tumor cells were observed in invasive component than that in the *in situ* component in 4 cases.

## Discussion

The purpose of the present study was to investigate ALDH1 expression in cancer cells in human breast cancer of different histologic stages. We found that ALDH1 was expressed in tumor cells in DCIS cases with a lowest rate, and in invasive cancer without EIC cases with a highest rate. Importantly, intratumoral ALDH1 expression in invasive component showed a higher rate of that in the *in situ* component in the same tumor for the first time. Furthermore, no significant association was observed between ALDH1 positivity and clinicopathological parameters in DCIS cases, while ALDH1 positive invasive breast cancers were significantly more likely to be with large tumor size, high grade, and high Ki67 expression.

In the present study, ALDH1 was expressed in tumor cells in 41.3 % of DCIS cases, 45.0 % of invasive cancer with EIC cases, and 93.1 % of invasive cancer without EIC cases. Charafe-Jauffret and colleagues [8] have found that ALDH1 was expressed in 34 % of inflammatory breast cancer with approximately 3 %-5 % positively stained cells in these tumors. However, Park and colleagues [9] have found that ALDH1 was expressed in few cases (less than 10 %) and no significant difference was observed in four histologic groups. To our knowledge, the difference of ALDH1 positivity in different studies may be due to the different cutoffs. In the study of Park [9], less than 10 % with ALDH1 stained was categorized as negative, while only 0 % was categorized as negative in other studies [1, 6, 8, 12]. The previous study [1] has shown that only 1 case was ALDH1 positive of 23 DCIS cases, while about 11 % invasive breast cancers were ALDH1 positive. Similarly, the expression of ALDH1 was lower in DCIS compared with IDC in our study. Moreover, one of the most interesting findings of our study is that intratumoral ALDH1 expression in invasive component showed a higher rate of that in the *in situ* component in the same tumor. All these findings may potentially implicate ALDH1 in the progression to invasion.

Morimoto and colleagues [1] have found that ALDH1 positive breast cancers are significantly more likely to be ER negative, PR negative, Her2 overexpression, and Ki67 positive, but ALDH1 is not significantly associated with poor clinical outcomes. Charafe-Jauffret and colleagues [8] have found that ALDH1 expression correlated with tumor grade, but there was no correlation with other clinical and pathologic features in inflammatory breast cancer. Moreover, previous studies [6, 8] have reported that ALDH1 is an independent prognostic marker to predict poor patient outcome in breast cancer. In our study, ALDH1 positive invasive breast cancers were significantly more likely to be with large tumor size, high grade, and high Ki67 expression, but no significant association was observed between ALDH1 positivity and clinicopathological parameters in DCIS cases. All the findings suggest ALDH1 positive cancer cells may be associated with aggressive phenotypes of breast cancer.

In this study, stromal ALDH1 positive cells were detected in most cases, but the frequencies of stromal ALDH1 positivity between benign breast disease and breast cancer were not significantly different. It should be pointed out that reports about human stromal stem cell in benign terminal ductal-lobular unit are limited [17–19]. Isfoss and colleagues [11] have described two morphologically distinct stromal ALDH1 positive cell types in benign mammary tissue from women with and without breast cancer. The most numerous and widely distributed stromal cell type is a spindle-shaped, fibrocyte-like cell with slender cytoplasm and a small, elongated nucleus, while the other ALDH1 positive stromal cell type shows a round or oval shape, with a relatively large nucleus. To the best of knowledge, the true identity of the stromal ALDH1 positive cell types is unclear and needs to be elucidated in future studies. In the present study, the high frequencies of stromal ALDH1 positivity among different stages of breast cancer were significantly different. Due to the small sample size, the difference may be attributed to chance. It seems that stromal ALDH1 expression may be involved in different processes in carcinogenesis, but future studies are needed.

Several limitations still exist in our study. First, the expression of ALDH1 in human breast cancer of different histologic stages was focused on in this study, and the prognosis of these cases was not available. Second, the sample size was relative small, and our findings should be confirmed in the future.

## Conclusions

In conclusion, ALDH1 may play an important role in the invasion of breast cancer, and may be associated with aggressive phenotypes of breast cancer. Future studies with large sample size should be needed to confirm our findings.

## Abbreviations

ALDH1: Aldehyde dehydrogenase 1
BT-ICs: Breast tumor-initiating cells
DCIS: Ductal carcinoma *in situ*
EIC: Extensive intraductal component
ER: Estrogen receptor
H&E: Hematoxylin and eosin
IDC: Invasive ductal carcinoma
IHC: Immunohistochemical
PR: Progesterone receptor
